# Trimethylamine functionalized radiation-induced grafted polyamide 6 fibers for *p*-nitrophenol adsorption

**DOI:** 10.1038/s41598-021-97397-y

**Published:** 2021-10-01

**Authors:** Shihab Ezzuldin M.Saber, Luqman Chuah Abdullah, Siti Nurul Ain Md. Jamil, Thomas S. Y. Choong, Teo Ming Ting

**Affiliations:** 1 Department of Chemical and Environmental Engineering, Faculty of Engineering, Universiti Putra Malaysia, UPM Serdang, 43400 Selangor Malaysia; 2 Institute of Tropical Forestry and Forest Products (INTROP), Universiti Putra Malaysia, UPM Serdang, 43400, Selangor Malaysia; 3 Department of Chemistry, Faculty of Science, Universiti Putra Malaysia, UPM Serdang, 43400, Selangor Malaysia; 4 Centre of Foundation Studies for Agricultural Science, Universiti Putra Malaysia, UPM Serdang, 43400, Selangor Malaysia; 5 Radiation Technology Division, Malaysian Nuclear Agency, 43000 Kajang, Selangor Malaysia; 6 North Refineries Company, Baiji, Salahuddin, Ministry of Oil Iraq

**Keywords:** Environmental sciences, Engineering, Materials science, Chemical physics, Nuclear physics, Chemical engineering, Environmental chemistry, Nuclear chemistry, Organic chemistry, Polymer chemistry, Surface chemistry

## Abstract

The method of pre-irradiation grafting was used with the aid of electron beam (EB) accelerator to accomplish the grafting of polyamide 6 fibers (PA6) with glycidyl methacrylate (GMA). The extent to which GMA was grafted on PA6 was found to be markedly influenced by the absorbed dose of radiation and the reaction time of grafting. Trimethylamine (TMA) was afterwards employed for the functionalization of GMA-grafted fibers (PA6-*g*-GMA). A range of analyses (e.g., FTIR, FESEM, XRD, BET, and pHpzc) were carried out to determine the physiochemical and morphological properties of the fibrous adsorbent. *p*-Nitrophenol (PNP) adsorption from aqueous solution was conducted with the resulting TMA-(PA6-*g*-GMA) adsorbent. The adsorption behaviour of PNP on the fibrous adsorbent was clarified by investigating the adsorption kinetics and isotherm. According to the results, the adsorption of PNP on TMA-(PA6-*g*-GMA) reflected the pseudo-second order model. Meanwhile, the isotherm analysis revealed that the best description of the equilibrium data was provided by Redlich–Peterson model, followed closely by Langmuir isotherm model. The achieved adsorption capacity was highest at 176.036 mg/g. Moreover, the adsorption was indicated by the thermodynamic analysis to be spontaneous and exothermic. Regeneration and recycling of the adsorbent was possible for a minimum of five cycles with no reduction in adsorption capacity. It was concluded that the fibrous adsorbent could have applications for the removal of PNP at industrial pilot scale.

## Introduction

Multifactorial problems related to environmental pollution have been uncovered and have worsened at global level owing to the fast pace at which urbanization and industrialization are proceeding^1^. Waterways are contaminated with a variety of highly toxic organic chemicals (e.g. pharmaceuticals, pesticides, phenols, dyes) generated by large-lot production and extensive use of different essential products. One of persistent organic pollutants (POPs) of extremely high toxicity that is released in waterways by the petrochemical, pesticide, explosive, dye, herbicide, and plasticiser industries is *p*-nitrophenol (PNP)^2^. Due to its toxicity and classification as a priority pollutant that cannot exceed 0.01–2.0 μg/L, PNP removal from waterways has received enormous attention^3^. However, in the case of the majority wastewater industrial facilities, basic physiochemical and biological treatment processes are not greatly effective at extracting PNP because the aromatic ring has a nitro (NO_2_) group that makes PNP more persistently stable and soluble in aqueous solutions^4^. Hence, the removing of PNP from wastewater is a pressing problem that must be addressed to mitigate its negative environmental and health impact.

Biodegradation^5^, oxidation^6^, membrane process^7^, and adsorption^8^ are among the wide range of methods adopted to remove of PNP from wastewater. Among these methods, the adsorption process is inexpensive, provides a high level of efficiency, is uncomplicated to perform, and permits recovery of both adsorbent and adsorbate^9^. For these reasons, it remains the methods typically employed, particularly for effluents with PNP in moderate-to-low levels. Earlier research has explored many adsorbents based on materials the purposes of contaminant adsorption. On the downside, adsorbent type and adsorption mechanism greatly influence the performance of the adsorption method. Therefore, novel adsorbents based on materials and possessing enhanced physiochemical qualities must be created. One separation method demonstrating high efficiency for contaminated effluent treatment is activated carbon^10^, but this method is quite expensive and presents post-use regeneration shortcomings. This calls for adsorbents that are affordable and straightforward to recycle^11^.

In the last decade, fibrous adsorbents have emerged as potential substitutes for conventional granular and powder adsorbents, being more cost-effective and demonstrating a highly active surface, suitable mechanical strength, capability of surface chemical change, reuse capability, and uncomplicated usage^12^. Hence, the development of such fibrous adsorbents to eliminate organic pollutants from aqueous solutions has attracted significant practical and academic attention. Among the various types of polymeric fibrous that can potentially serve as adsorbents for removal of different contaminants, including polyethylene (PE), polypropylene (PP), polyacrylonitrile (PAN), polyamide 6, and polylactic acid (PLA). A major engineering polymeric fiber, polyamide 6 (PA6) displays exceptional mechanical properties and is resistant to mild acids and alkalis, highly stable thermally, and cost-effective^13^. It is typically subjected to physical or chemical modification according to whether the modification process yields novel chemical bonds^14^.

Due to their robust affinity for certain compounds (e.g. phenols), adsorbents based on materials and comprising specific functional groups (e.g. amine group) have been suggested to have high efficiency as adsorbents^15^. From this perspective, an alternative strategy is supplied by the method of graft polymerization, which is frequently used in adsorption applications to produce selective surfaces with well-regulated properties and therefore has attracted the interest of researchers^16^. Various techniques devised to improve the surface characteristics of fibrous adsorbents via graft polymerization including chemical grafting^17^, photo grafting^18^, and high-energy radiation^19^. Radiation-induced graft polymerization (RIG) (i.e. high energy radiation) is straightforward, flexible with regard to regulation of the quantity of integrated side-chain grafts, and does not require chemicals for reaction commencement^20^. Therefore, it is a particularly notable method. Furthermore, it can greatly improve the adsorption process by enabling the preparation of new functionalized fibrous forms and qualities that are better than other, costlier and environmentally damaging methods. Application of RIG in the emulsion phase attenuates environmental impact based on the use of a monomer mixture and water. Such an approach does not require toxic organic solvents, is affordable because it employs reduced levels of irradiation and monomers (reduce monomer consumption), and it affords a higher degree of grafting^21^.

The grafted monomer on PA6 that was chosen for the purposes of the present research was GMA owing to the possibility of modification of its epoxy group into a range of functional groups (e.g. amine, carboxylic groups). The addition of functional groups (e.g. primary, secondary, and tertiary amines) is facilitated by the straightforward immobilization of amine groups on the GMA-grafted substrates through a ring-opening reaction. Furthermore, various functional group or ionic moieties appropriate for different uses can be introduced as a result of the opening of the epoxy ring under mild conditions of reaction^22^. Moreover, as an affordable reagent, GMA is extensively employed to produce epoxy-functional methacrylic resins (e.g. precursors for the manufacture of coatings and adhesives) on an industrial scale^23^.

Development of an efficient adsorbent from cost-effective fibrous material was the goal of this research. To that end, GMA was grafted on PA6 via radiation-induced emulsion grafting to produce (PA6-*g*-GMA) fibers. Trimethylamine (TMA) was subsequently used for the chemical functionalization of the grafted fibrous material to improve its surface-active sites. PNP adsorption from aqueous solution was conducted for additional assessment of how TMA-(PA6-*g*-GMA) fibrous adsorbent performed, which no other research has done so far. Moreover, several adsorption variables were analysed, such as adsorbent dose, pH of PNP solution, temperature, initial PNP concentration and contact time. To understand how the PNP was adsorbed, kinetic and equilibrium data were analysed as well.

## Experiment

### Materials and methods

The fibrous adsorbent was prepared on a substrate of polyamide 6 fibers acquired from Reliance Sdn Bhd (Malaysia). Glycidyl methacrylate (C_7_H_10_O_3_) (purity of 97%) and Tween-20 (Tw-20) surfactant were obtained from Sigma-Aldrich (Saint Louis, MO, USA), while trimethylamine (40% solution) and industrial grade propanol were sourced from Merck (Darmstadt, Germany). Millipore Direct-Q™ water deioniser facilitated production of deionised water DI. The modelled organic pollutant that was employed was *p*-nitrophenol (C_6_H_5_NO_3_) (Acros Organics, New Jersey, USA). The chemicals were not treated further but used as received.

### Methods

#### Irradiation of PA6 fiber substrate

PA6 fibers were placed in polyethylene zipper bags that had been deaerated with nitrogen gas to prevent any oxygen contact with the samples. Subsequently, the bags with the samples were put in a tray on dry ice, which was transferred to the irradiation chamber via a conveyor and irradiated with an electron beam (EB) accelerator (NHV-Nissin High Voltage, EPS3000). The conditions of accelerator operation were 1 MeV acceleration energy, 10 mA beam current, and 1–20 m/min conveyor speed. The dose of radiation administered to the samples varied in the range of (10–50) kGy.

#### Grafting process

The grafting system presented in^24^ was applied to perform the grafting reaction. The first step was weighing and placing the irradiated samples in glass ampoules with evacuation for half an hour. This was followed by 10-min emulsification of the glass vessels with a content of 5% GMA monomer and 0.5% Tw-20 solutions with distilled water. Deaeration was performed next via half-hour bubbling with purified nitrogen gas. A tri-way stopcock facilitated the transfer of the GMA monomer solutions to the irradiated samples in the evacuated glass ampoules. After sealing, the glass ampoules were immersed in a water bath with a temperature of 40 °C for intervals of between 20 and 180 min. Upon completion of the grafting reaction, the grafted fiber was taken out, immersed, washed several times with propanol, and oven-dried at 50 °C.

The grafting yield had to be obtained through gravimetry based on the formula below:

1 $${\text{GY}}\% = \frac{{{\text{W}}_{{\text{f}}} - {\text{W}}_{{\text{i}}} }}{{{\text{W}}_{{\text{i}}} }} \times 100.$$

In the above, the pre-grafting or initial sample weight is denoted by W_i_ and the post-grafting or final sample weight is denoted by W_f_.

#### Functionalization of the irradiated grafted fibers

Functionalization of the PA6-*g*-GMA was achieved by treating it with TMA to incorporate the amine group on the fibrous adsorbent surface so as to be able to modify the irradiated grafted fibers. Preparation of 40-g solution involved mixture of TMA solution in deionised water at a ratio of (10% TMA: 90% deionized water) in two neck round bottom flasks operated under reflux at 80 °C in water bath (“Daihan” Wise Bath^®^ WB Digital Precise Water Bath). After the transfer of grafted sample to the TMA solution, reflux was performed for 180 min at 80 °C. The next step was TMA-functionalized (PA6-*g*-GMA) formed was repeatedly washed with methanol and 24-h oven-drying at 50 °C. The formula below was applied to obtain the TMA amine group on the functionalized grafted fibers:

2 $$AG = \frac{{{\text{W}}_{{{\text{Mg}}}} - {\text{W}}_{{\text{g}}} }}{{{\text{W}}_{{{\text{Mg}}}} }} \times \frac{1000}{{MW}} .$$

**Figure 1:**
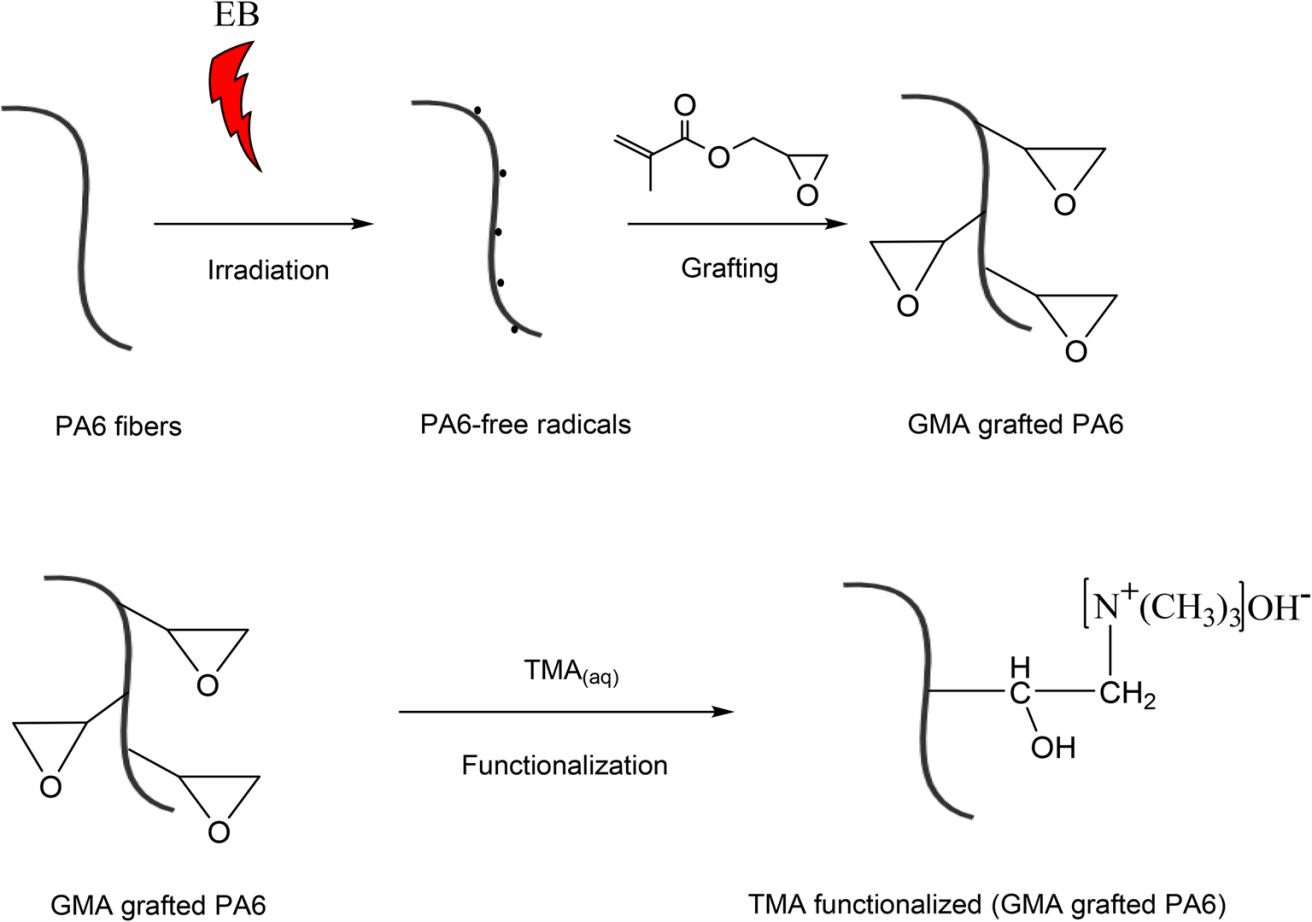
An illustrational mechanism for the fibrous adsorbent preparation by grafting of GMA onto PA6 and subsequent functionalization with TMA.

In the above, the amine group is denoted by AG, the dry weight of the TMA-functionalized sample and the dry weight of the grafted sample are denoted by W_Mg_ and W_g_ respectively, while the TMA molecular weight is denoted by MW (59.11 g/mol)^21^. Figure 1 illustrates the pathways of radiation, grafting, and functionalization.

### Fibrous adsorbent characterization

Fourier transform infrared spectrometer (FTIR) (1750X-Perkin Elmer Inc., Waltham, MA, USA) was employed to characterize the fibrous absorbent prior to and after functionalization, while the crystal structures of the samples were analysed with the help of an X-ray diffractometer (XRD) (model XRD-6000, Shimadzu, Japan). The Brunauer–Emmett–Teller (BET) surface area, pore volume of the samples was measured using porosimeter (Micromeritics Instrument Corporation, Model-3Flex, Norcross, GA, USA). Furthermore, field emission scanning electron microscopy (FESEM, Nova NanoSEM 230, FEI, USA) enabled analysis of sample morphology under operation conditions of 5-kV acceleration voltage, 1000 × magnification, and 100 μm resolution. To achieve images of high resolution, sputter coater was employed for coating the samples with a thin Pt layer. The approach recommended in^25^ was applied to determine the pH of TMA-(PA6-*g*-GMA) at the point of zero charge (pHpzc).

### Adsorption experiments

PNP adsorption on TMA-(PA6-*g*-GMA) was examined through batch experiments. Distilled water was used for preparation of a stock of PNP solution of 1 g/L and dilution of solution aliquots was performed to achieve the necessary concentrations in the range of (20–200) mg/L prior to initiating the adsorption experiments. All volumetric flasks containing PNP were wrapped with aluminium foil to avoid photo-oxidation. A set of conical flasks with a volume of 250 mL were employed for the experiments and those were placed in a temperature-regulated incubator shaker (ST-200R, SASTEC) with 150-rpm shaking speed and covered with black fabric throughout the experiments. The one variable at a time (OVAT) method was applied to attain the optimum outcomes. The effect of fibrous adsorbent dose on PNP adsorption was investigated by using different doses of TMA-(PA6-*g*-GMA) in the range of (0.02–0.2) g. Furthermore, to assess pH impact on adsorption, 0.1 M of NaOH solution and 0.1 M of HCl solution were used for adjustment of PNP solution pH from 3 to 11. The influence of temperature of adsorption performance was evaluated through variation of temperature in the range between 25 and 45 °C. At pre-established times, the aliquot of the sample solution was taken out, filtered, and the PNP concentration was calculated by analyzing the UV absorbance at 317 nm by using UV–vis spectrophotometer (Shimadzu UV-1800, Japan).

Five cycles of sorption and desorption were performed to analyse fibrous adsorbent regeneration. 15 mL of 0.1 M of NaOH was used for elution (desorption) of 0.05 g PNP loaded TMA-(PA6-*g*-GMA), followed by washing of the regenerated fibrous material and 24 h drying in the oven at 50 °C.

### Data analysis

The results were the average of three experimental iterations. The formulas below were applied to determine the results for the PNP removal and adsorbed amount.

3 $$RE = \frac{{{\text{C}}_{{\text{o}}} - {\text{C}}_{{\text{e}}} }}{{{\text{C}}_{{\text{o}}} }} \times 100,$$

4 $$q_{e} = \frac{{({\text{C}}_{{\text{o}}} - {\text{C}}_{{\text{e}}} ) {\text{V}}}}{{\text{m}}}.$$

In the above, the initial PNP concentration (mg/L) is denoted by C_o_ and the equilibrium PNP concentration (mg/L) is denoted by C_e_, while the volume of PNP_(aq)_ solution (L) and the adsorbent mass (g) are denoted by V and m, respectively.

Kinetic studies in the context of adsorption was investigated based on nonlinear models of pseudo-first order^26^, pseudo-second order^27^, Elovich^28^, and intraparticle diffusion^29^. The kinetic models were defined according to the nonlinear equations below.

Pseudo-first order:

5 $${\text{q}}_{{\text{t}}} = q_{e} (1 - {\text{ e}}^{{ - {\text{K}}_{1} {\text{t}}}} ).$$

Pseudo second order:

6 $${\text{q}}_{{\text{t}}} = \frac{{{\text{q}}_{{\text{e}}}^{2} {\text{K}}_{2} {\text{t}}}}{{1 + {\text{q}}_{{\text{e}}} {\text{K}}_{2} {\text{t}}}}.$$

Elovich:

7 $${\text{q}}_{{\text{t}}} = \frac{1}{{\text{b}}}\ln (1 + {\text{abt}}).$$

Intra-particle diffusion:

8 $${\text{q}}_{{\text{t}}} = {\text{K}}_{{{\text{ip}}}} {\text{t}}^{0.5} + {\text{C}}_{{{\text{ip}}}} ,$$

where, the reaction time is denoted by t (min), while the adsorption rate constant for the pseudo-first order model is denoted by K_1_ (1/min) and the adsorption rate constant for the pseudo-second order model is denoted by K_2_ (g/mg min). In the Elovich equation, the initial adsorption rate constant (mg/g min) is denoted by a, while the desorption rate constant (g/mg) is denoted by b. In the intra-particle diffusion equation, the intra-particle diffusion rate constant is given by K_IP_ (mg/g min^0.5^), the adsorption quantity of adsorbate by the adsorbent at the equilibrium condition is given by q_e_, while at time t is given by q_t_.

For isotherm analysis, PNP concentrations in the range (20–200) mg/L were used at equilibrium conditions. Langmuir^30^, Freundlich^31^, Temkin^32^, and Redlich–Peterson^33^ nonlinear isotherm models were used with the equilibrium data to determine the model constants. The associated nonlinear equations are provided below.

Langmuir model:

9 $$q_{e} = \frac{{q_{max} K_{L} C_{e} }}{{1 + K_{L} C_{e} }}.$$

Freundlich model:

10 $$q_{e} = K_{F} C_{e}^{1/n} .$$

Temkin model:

11 $$q_{e} = \frac{RT}{{b_{T} }}\ln (k_{T} C_{e} ).$$

Redlich Peterson model:

12 $$q_{e} = \frac{{K_{RP} C_{e} }}{{1 + \alpha_{RP} C_{e}^{\beta } }},$$

where, the maximum adsorption capacity is denoted by *q*_*max*_ (mg/g), the residual PNP concentration at adsorption equilibrium is denoted by C_e_ (mg/L), the parameter associated with adsorbent surface inhomogeneity is denoted by n, the universal gas constant of 8.314 J/mol. K is denoted by R, the absolute temperature is denoted by T (K), the binding site affinity is denoted by $\alpha_{RP}$, the isotherm exponent is denoted by β, the Langmuir parameter is denoted by K_L_ (L/mg), the Freundlich parameter is denoted by K_F_ (mg/g)(L/mg)^1/n^, the Temkin parameters are denoted by K_T_ (L/mg) and b_T_ (J/mol), and the Redlich–Peterson parameter is denoted by K_RP_ (L/g).

Moreover, the character of the adsorption process was established based on the equilibrium parameter R_L_, which represented a dimensionless constant and was given by the equation below.

13 $${\text{R}}_{{\text{L}}} = \frac{1}{{1 + {\text{K}}_{{\text{L}}} {\text{C}}_{{\text{o}}} }},$$

where, the initial PNP concentration (mg/L) is denoted by C_o_ and the Langmuir adsorption constant (L/mg) is denoted by K_L_.

The regenerative performance of the fibrous adsorbent was determined through the following equation:

14 $$Reg.\% = \frac{{q_{{e{\text{-}}reg.}} }}{{q_{{e{\text{-}}fresh}} }} \times 100.$$

### Regression analysis

The discrepancies between the experimental data and the theoretical data derived from the models employing the Solver Add-ins for Microsoft Excel spreadsheets were assessed based on the correlation coefficient (*R*^2^) and the sum of square errors (SSE). The purpose of this was to determine the model that fitted the data most closely. The formulas for calculating the *R*^2^ and SSE are provided below.

15 $$R^{2} = \frac{{\sum (q_{m} - q_{cal} )^{2} }}{{\sum (q_{cal} - q_{m} )^{2} + \sum (q_{cal} - q_{\exp } )^{2} }},$$

16 $$SSE = \mathop \sum \limits_{1}^{n} (q_{\exp } - q_{cal} )^{2} ,$$

where, the average value of the experimental adsorption capacity is denoted by *q*_*m*_ (mg/g), the experimental adsorption capacity is denoted by *q*_*exp*_ (mg/g), and the determined adsorption capacity is denoted by *q*_*cal*_.

## Results and discussion

### RIG of GMA on PA6 and TMA functionalization

**Figure 2:**
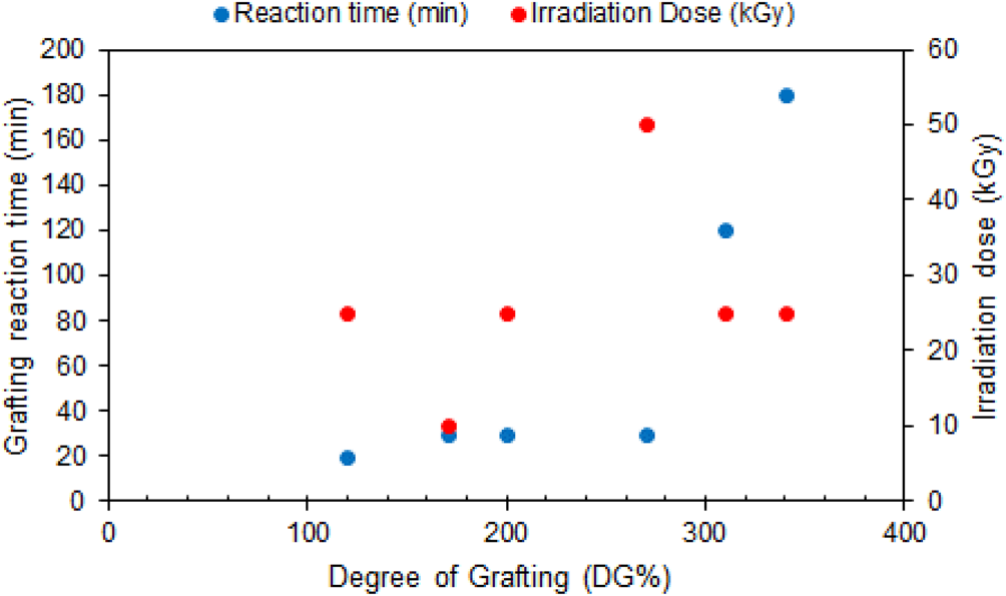
Effect of different irradiation dose and graft reaction time on degree of grafting of GMA on PA6 (Monomer concentration (5%) TW20 (0.5%), at 40 °C).

The degree of grafting courses for RIG of GMA on PA6 fibers substrate at different reaction times and absorbed doses is indicated in Fig. 2. In general, the increase in the degree of grafting (DG) is proportional to the increase in reaction time. The longer the reaction time, the more likely the monomer molecules are to diffuse to the initiation sites and propagate polymer chains, resulting in increasing the degree of grafting^21^. Hence, extension of the reaction time from 20 to 180 min resulted in a corresponding increase in DG% from 120 to 340%, as can be seen in Fig. 2.

Chemically initiated grafting requires a chemical initiator to generate radicals on the polymer chain and allow graft polymerization of a monomer onto the polymer. However, most chemical initiators display toxicity and are typically left as residues that can leach out of the synthesized material. The outcome of standard polymerization can be accomplished via RIG whilst avoiding chemical initiators. To determine the impact of absorbed doses, free radicals are produced when radiation (e.g., gamma radiation, electron beam) interacts with the trunk polymer. The diffusion and polymerization of monomer molecules subsequently occur on those free radicals. This approach implies that irradiation of trunk polymers at elevated absorbed dose ought to result in higher DG materials. The proliferation of existing radicals in the polymer substrate as the dose increases and the fact that they often participate in reaction activation are the reasons for the DG% increase^34^. Figure 2 illustrates this association as well as the impact of absorbed dose on DG. In the present study, it has been attempted to reduce the reaction time and achieved a greater degree of grafting. Samples subjected to irradiation at an absorbed dose of 25 kGy made it possible to reach the 200% target DG at a shorter reaction time.

The electron beam was used to graft GMA on PA6 fibers. Through energy transfer to molecules in the PA6 fibers backbone, high-energy electrons bombard the fibers, resulting in the formation of free radicals (Fig. 1). This allows the addition of the GMA monomer to the PA6 backbone with active radicals. The grafting reaction on PA6 chains can start instantly via the double bond occurring in GMA because the active radicals display high reactivity towards GMA monomers within the system^35^. The monomer-fiber covalent bond that thus forms mediates propagation of grafting on the irradiated fibers^36^.

The epoxy group present in the GMA chemical structure can serve as precursor for binding with other reactive groups. Epoxy ring opening in GMA underpins the emergence of novel functional groups. A covalent bond forms in the functionalization stage due to the attachment of TMA molecules to the epoxy groups on the grafted chains of PA6 fibers. At the end of the functionalization, the yield of amine density was calculated to be 2.843 mmol/g.

### Fibrous adsorbent physiochemical properties

#### FTIR spectral analysis

**Figure 3:**
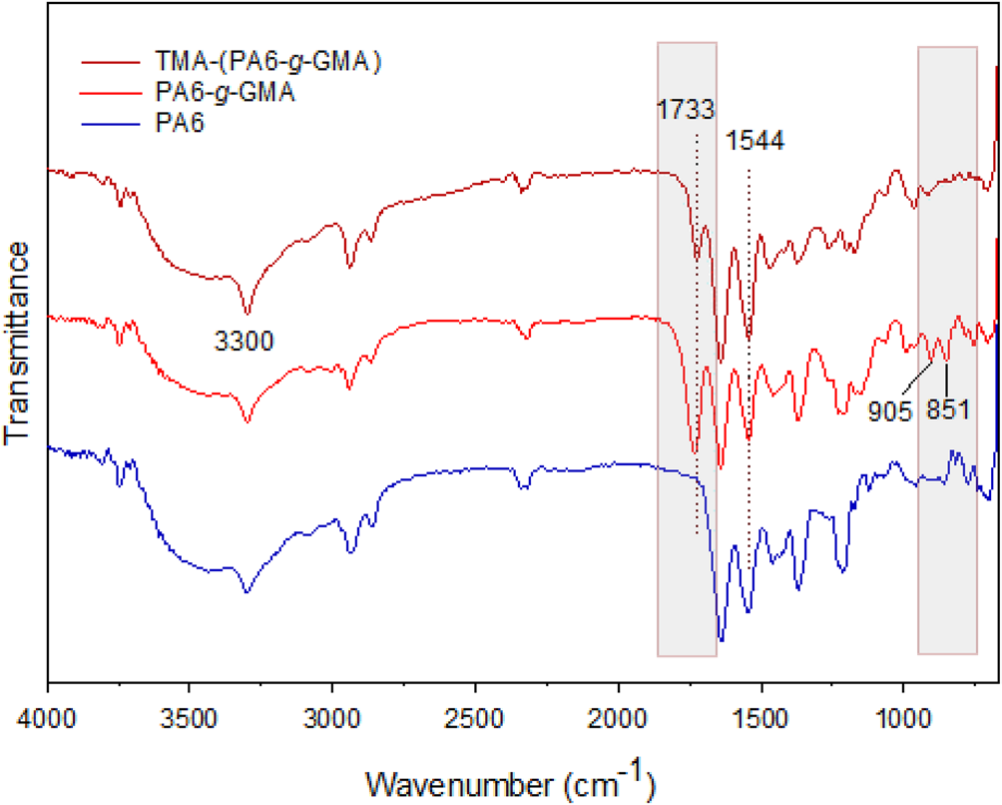
FTIR spectra of pristine PA6 fibers, PA6-*g*-GMA (200% DG), and TMA-(PA6-*g*-GMA).

The FTIR spectra of the original PA6, GMA grafted, and obtained TMA functionalized fibers are illustrated in Fig. 3. Unaltered PA6 fibers exhibited peak at 3300 cm^−1^ corresponding to hydrogen-bonded NH stretching, at 2936 cm^−1^ corresponding to CH_2_ asymmetric stretching, and at 2858 cm^−1^ corresponding to CH_2_ symmetric stretching^24^. Meanwhile, CH_2_ scissoring vibration was reflected by a band at 1460 cm^−1^ and CH_2_ twisting vibration was reflected by a band at 1370 cm^−1^^37^. A band at 1639 cm^−1^ was associated with amide I, while a band at 1544 cm^−1^ was associated with amide II; these displayed a minor reduction due to GMA grafting. Furthermore, C=O stretching vibration was reflected by the emergence of a new peak at 1733 cm^−1^, while the occurrence of grafted GMA epoxy group was signalled by stretching vibration peak at 905 cm^−1^ and asymmetrical expansion at 851 cm^−1^. This strongly supports GMA graft inclusion on PA6 fibers^23^. Moreover, the success of the opening of the epoxy ring and TMA amine incorporation were reflected by the spectrum related to the fibrous adsorbent with TMA content, which was highly distinct from the spectrum of the original and grafted PA6 as attested by the vanishing of stretching accountable for GMA epoxy group (Fig. 3). In addition, the inclusion of –OH following ring opening gave rise to the broadband at 3300 cm^−1^, whilst –NH peaks at 1643 cm^−1^ and peak intensity increase at 1544 cm^−1^ could be attributed to the –N^+^(CH_3_)_3_ moieties which confirm the presence of TMA after functionalization reaction^38^.

#### XRD analysis

**Figure 4:**
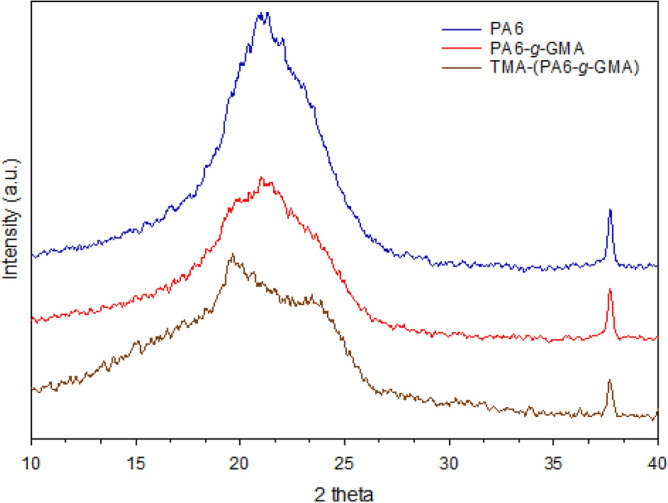
XRD patterns of original PA6, PA6-*g*-GMA and TMA-(PA6-*g*-GMA).

Figure 4 presents the diffractograms associated with original PA6, GMA-grafted PA6, and TMA-functionalized grafted PA6. XRD analysis was conducted to identify how grafting and amination altered the PA6 crystalline structure. The crystallite size (L) of PA6, grafted, and TMA functionalized grafted PA6 was compared to undertake intercalation based on the Scherrer formula. The equation applied is provided below.

17 $$L = \frac{k \lambda }{{B\cos \theta }}.$$

In the above, the crystallite size is denoted by *L*, the diffraction angle is denoted by θ, a dimensionless shape factor with standard value of around 0.9 is denoted by *k*, the line broadening at half the maximum intensity is denoted by *B*, while the Cu Kα radiation wavelength (1.5406 Å) is denoted by λ. The structure of a semicrystalline polymer with crystallinity of a form reflected by the peak at 2θ = 21.05° was revealed by the diffractogram of the initial PA6. Crystallinity diminished, as indicated by decline in peak intensity with wider peak at 21.34°, when GMA was grafted on PA6. Equation (17) was applied and produced values of 10.26 nm for PA6 crystallite size and 8.88 nm for the PA6-*g*-GMA crystallite size. The grafting of vinyl benzyl chloride on nylon fibers was found to produce a comparable decline in peaks as well^39^. The cause was a decrease in grafted fiber crystallinity due to dilution with amorphous P(GMA) grafts and potential partial crystallite disruption. It is noteworthy that, following TMA functionalization of the grafted PA6, the diffractograms showed a peak associated with crystallite conversion from 2θ = 21.34° into 2θ = 19.61°. Correspondingly, the crystallite would have a size of 12.80 nm. This suggested the homogeneous distribution of TMA units on the surface.

#### Features of fibrous adsorbent morphology

**Figure 5:**
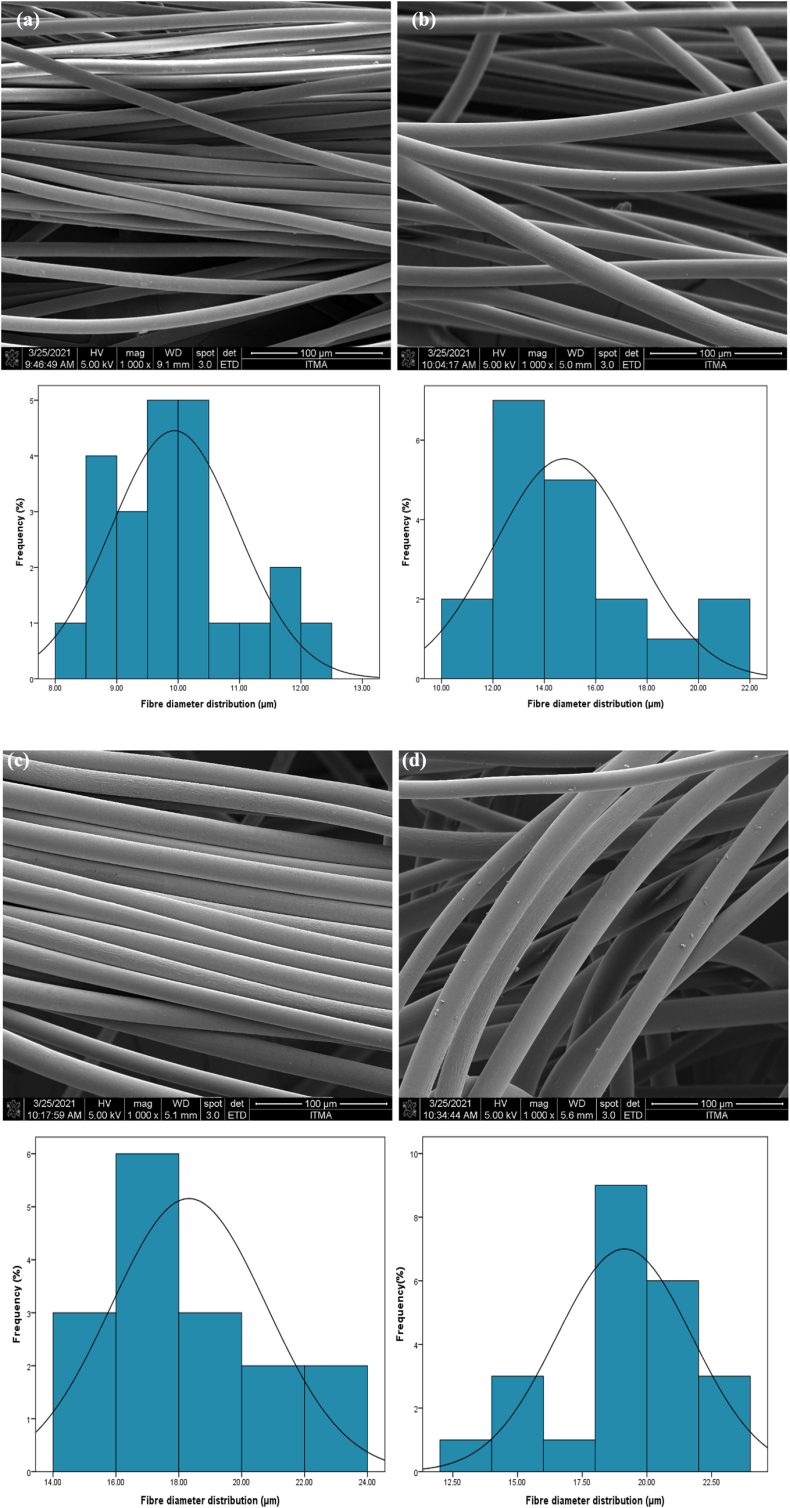
FESEM images of original PA6 (**a**), PA6-*g*-GMA (**b**), TMA-(PA6-*g*-GMA) (**c**), PNP loaded TMA-(PA6-*g*-GMA) (**d**).

FESEM analysis was conducted to investigate the alterations in morphology in PA6, grafted, and TMA-grafted PA6 fibers surface. Figure 5 illustrates the related micrographs. Based on the FESEM analysis of individual fibers, ImageJ software was applied to measure the microsphere diameters and fiber size distributions (ImageJ 1.53e)^40^. As can be observed, P(GMA) inclusion led to an increase in PA6 fibrous average diameter from 9.94 μm (Fig. 5a) to 14.78 μm (Fig. 5b). GMA grafting was verified by the images, which also indicate the uniform distribution on the surface. The “coating nature” of grafting to the surface, determining an increase in fiber diameter from 14.78 μm (Fig. 5b) to 18.32 μm (Fig. 5c), was indicated when the grafted fiber was subjected to amination. Furthermore, the PA6 fiber was not visibly damaged using a low absorbed dose during GMA grafting.

#### Surface area analysis

**Figure 6:**
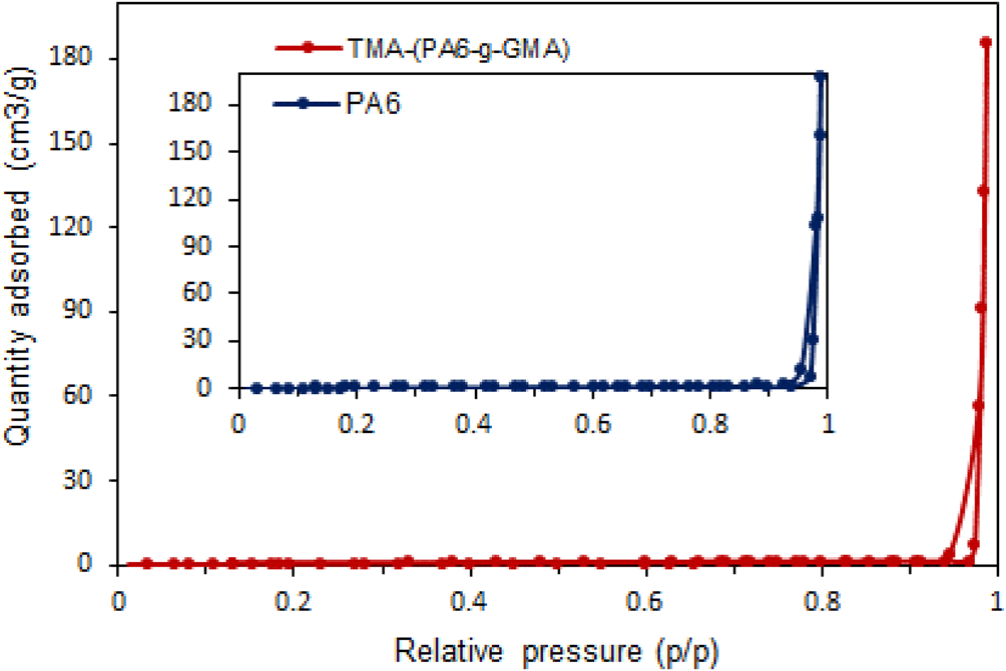
N_2_ adsorption–desorption isotherm of PA6 (blue) and TMA-(PA6-*g*-GMA) (red).

Additional analysis of adsorbent pore parameter was conducted by measuring the nitrogen sorption. More specifically, N_2_ adsorption–desorption isotherms were used for measurement of the original PA6 and TMA-(PA6-*g*-GMA) in terms of specific surface area (SSA), volume and size of pores. Figure 6 illustrates the corresponding isotherms. The occurrence of mesopores of (2–50) nm was deduced from the standard type IV adsorption–desorption isotherm exhibited by the adsorbents as per the IUPAC classification^41^. The effect of TMA functionalization on SSA, volume and size of the pores of PA6 fibrous adsorbent is detailed in Table S1. Comparison with the untreated adsorbent regarding SSA revealed that the pore volume diminished following GMA grafting and TMA treatment, whereas the pore diameter expanded following TMA treatment. This pointed to the coating of the fibrous surface with a film-like shell produced by the grafted GMA, which decreased the SSA by partially filling the spaces among microfibrils. The success of the grafting process all through the fiber surface seems to be confirmed by such findings and is further supported by FESEM analysis, which showed a marked increase in the average diameter of grafted and aminated fibrous adsorbent compared to untreated PA6. Earlier research indicated that the alterations caused a decrease in SSA such as GMA grafting on cellulosic fibers^42^. Therefore, it was anticipated that the expansion in fiber diameter would reduce the SSA of the fibrous adsorbent.

### Factors affecting *p*-nitrophenol adsorption

#### The impact of adsorbent dose on PNP adsorption

**Figure 7:**
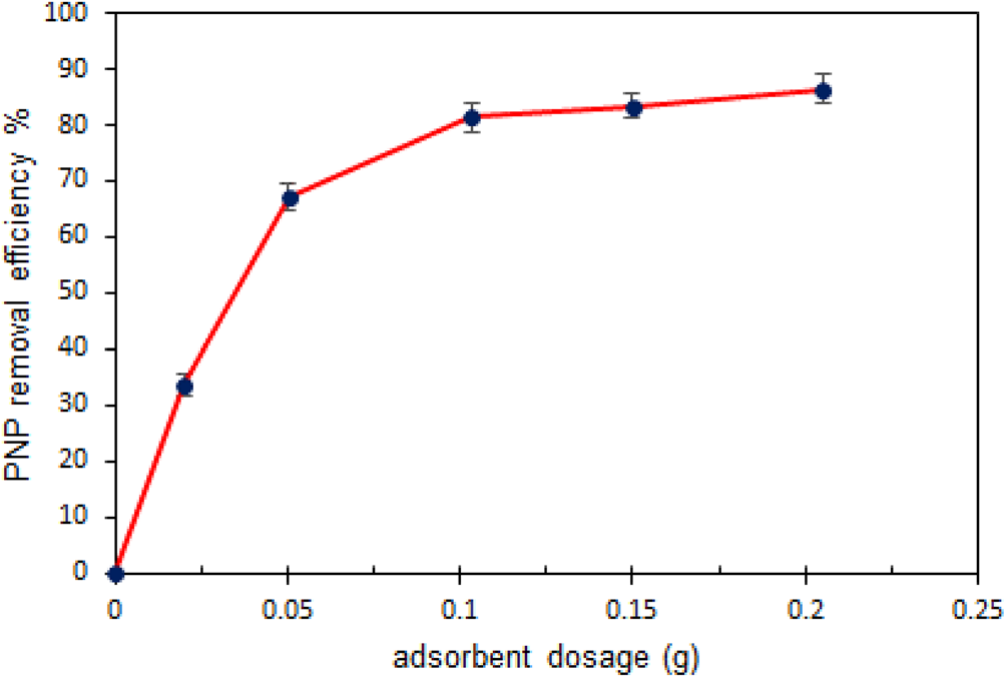
Effect of adsorbent dosage to the PNP removal efficiency (PNP initial concentration: 50 mg/L; time: 1 h; temperature 298 K and 150 rpm).

The adsorbent dose was varied between (0.02–0.2) g to assess the PNP removal yield. The proportion of PNP removal increased from 33.75 to 81.31% when the adsorbent dose was increased from 0.02 to 0.1 g (Fig. 7), which can be explained in terms of the fact that the contact surface area of the fibrous adsorbent expanded and more vacant active sites became available^43^. However, PNP removal did not improve markedly when the adsorbent dose was increased at values higher than 0.1 g because adsorption sites clustered together or overlapped so that the overall adsorption surface area accessible to PNP molecules diminished. Hence, by considering the adsorption impact and economic expenditure, it was established that 0.1 g of TMA-(PA6-*g*-GMA) was the optimum adsorbent dose. An earlier study obtained comparable results regarding the use of biomass material for adsorption of organic water pollutant ^44^.

#### The impact of solution pH on PNP adsorption

**Figure 8:**
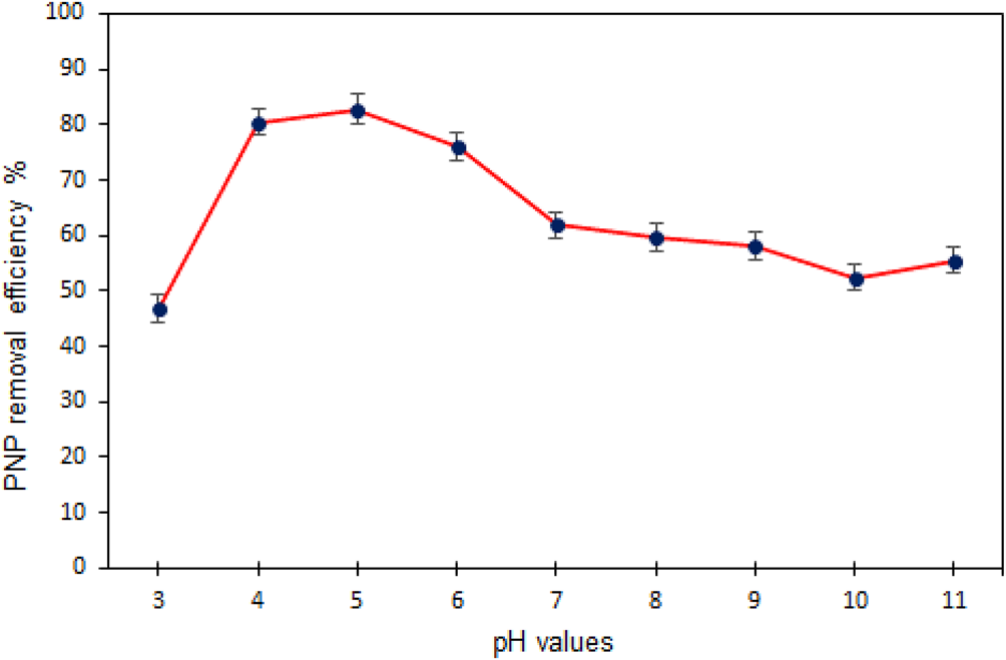
Effect of the solution’s pH on PNP adsorption by TMA-(PA6-*g*-GMA) at different pH values (50 mg/L initial concentration of PNP, 0.1 g/100 mL adsorbent dosage, temperature 298 K, time 1-h and 150 rpm).

By impacting the degree of pollutant ionization and adsorbent surface properties, solution pH is a major process variable with implications for adsorbent-based removal of pollutants^45^. To gain insight into the behaviour of PNP in solution, the level of pH was varied between 3 and 11 to analyse the impact of initial pH on PNP adsorption onto TMA-(PA6-*g*-GMA). While, other variables were kept constant, with adsorbent dose of 0.1 g, 50 mg/L PNP concentration, 1 h contact time, and 150 rpm shaking speed. Figure 8 illustrates how PNP adsorption was affected by the pH of solution; an increase in pH from 3.0 to 4.0 determined an increase in PNP removal from 46.79 to 81.04%. A pH of 5.0 was associated with maximum removal of PNP, which subsequently declined gradually at higher pH levels towards the alkaline medium. The pHpzc of TMA-(PA6-*g*-GMA) helps to clarify why PNP uptake varied according to solution pH. Figure S1 shows that the pHpzc of TMA-(PA6-*g*-GMA) was 9.1 ± 0.1; when the solution pH was lower than the pHpzc, the solution tends to donate more protons than hydroxide groups. This led to more positively charged sites emerging on the surface of the adsorbent, which intensified the electrostatic attraction between the fibrous adsorbent surface with a positive charge and the PNP molecules with a negative charge. As a result, removal became more efficient. On the other hand, when the pH was higher than the pHpzc, there was a reduction in the adsorbent surface charge, yielding a partial negative charge that made PNP removal less efficient^46^.

#### Temperature impact on PNP adsorption

**Figure 9:**
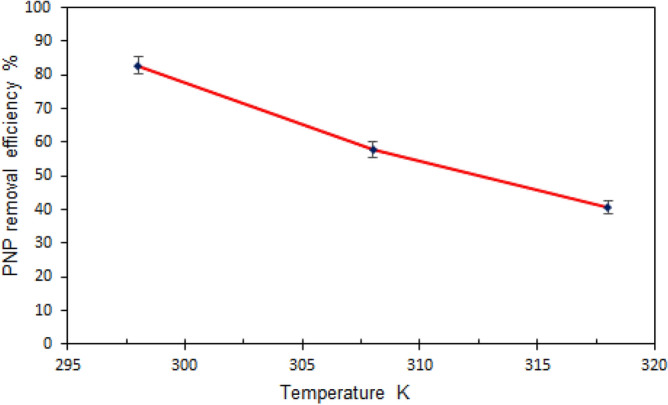
Effect of temperature on PNP removal efficiency by TMA-(PA6-*g*-GMA) (adsorbent dose: 0.1 g/100 mL; PNP initial concentration: 50 mg/L, pH 5, time: 1 h and 150 rpm).

Given that the physisorption and chemisorption processes depend to a great extent on temperature, this parameter was investigated as well. In turn, the physical and chemical qualities of the adsorbent and adsorbate as well as the reaction type determine the impact of temperature, regardless of whether it is favourable or unfavourable. In general, temperature rise causes an increase in the rate of endothermic chemical reactions, whereas temperature and exothermic reactions are inversely proportional^47^. In this research, three temperatures (i.e., 298, 308, and 318 K) were employed to assess the impact of temperature. The optimum adsorbent dose of 0.1 g and pH of 5 were used to conduct the experimental work. It was found that PNP adsorption was negatively affected by temperature, with maximum PNP removal of 82.81% onto TMA-(PA6-*g*-GMA) being achieved at lower temperature. As the temperature increased to 318 K, PNP removal diminished (Fig. 9). This was considered to reflect the exothermic character of the process of adsorption, since in most cases of exothermic adsorption functions, temperature increase causes pollutant desorption to the fluid phase at equilibrium. To put it differently, elevated temperature increased the diffusive mass transfer and PNP solubility in water and reduced the strength of adsorptive forces between adsorbent sites and adsorbate. Consequently, the accessibility and affinity between the adsorbent and PNP declined, making removal less efficient^48^. An earlier study reported similar observations for the use of a polymer-supported ionic liquid adsorbent to remove PNP^15^. Owing to the findings obtained, additional adsorption analysis was conducted in the present research at 298 K.

#### The impact of initial PNP concentration and contact time

**Figure 10:**
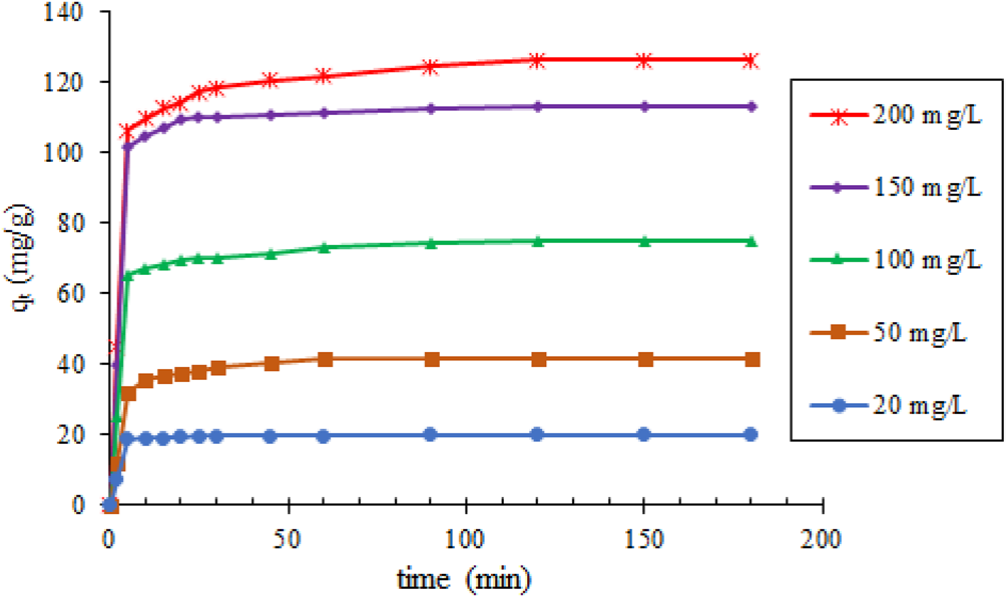
Effect of contact time and initial PNP concentration on the adsorption of PNP by TMA-(PA6-*g*-GMA) (0.1 g adsorbent dose, pH 5, 298 K, and 150 rpm agitation speed).

Five initial PNP concentrations between (20 and 200) mg/L were examined in terms of adsorption efficiency in relation to time in order to assess how the adsorption equilibrium was affected by contact time and initial PNP concentration. No changes were made to the other parameters of experiment, including adsorbent dose (0.1 g), pH (5), and temperature (298 K). The manner in which the PNP concentration changed with time was kept track of and Eq. (4) was applied to compute the PNP adsorbed on TMA-(PA6-g-GMA) (*q*_*e*_). It was found that PNP was quickly adsorbed on the TMA-(PA6-*g*-GMA) surface across the first 5 min and the overall PNP adsorption was higher when contact time increased but became fixed when contact time became equivalent to equilibrium time (Fig. 10). The fast and efficient adsorption of PNP molecules was interpreted by the numerous active functional group sites that are normally present in TMA-(PA6-*g*-GMA). Furthermore, the proportion of PNP adsorption increased as the initial PNP concentrations increase, as revealed by the graphical illustration in Fig. 10. One explanation for this is that the elevated concentration gradient mobilized the PNP molecules in the direction of active adsorption sites, overcoming the mass transfer resistance (i.e. resistance from the water phase to the adsorbent surface) across the PNP solution and surface of the adsorbent^8^. An earlier study obtained comparable results regarding the initial concentration of phenol adsorption on porous carbon from *Toona sinensis* leaves^49^.

### Adsorption process analysis

#### Kinetics of adsorption

The type of reaction related to the PNP adsorption, the rate of PNP removal, and the manner in which PNP diffused onto TMA-(PA6-*g*-GMA) were derived from the outcomes of kinetics experiments work. To determine how the developed fibrous material adsorbed PNP, pseudo-first order (Eq. 5), pseudo-second order (Eq. 6), Elovich (Eq. 7), and intraparticle diffusion (Eq. 8) models were employed to fit the adsorption kinetic data and the findings are presented in Figure S2(a–d) and Table 1. Given the high correlation coefficient *R*^2^ and the low SSE values, the kinetic data pertaining to the fibrous adsorbent samples were well-fitted by the models. Maximal *R*^2^ (> 0.99) and minimal SSE values were achieved when the pseudo-second order kinetic model was used to fit the kinetic data; this model also yielded adsorption amount (*q*_*cal*_) values close to the experimental values (*q*_*exp*_). According to the premises associated with this model, it was deduced that the surface activity sites contained in the fibrous adsorbent influenced the rate of PNP adsorption onto TMA-(PA6-*g*-GMA) and that chemisorption controlled the process of adsorption, involving valence forces via electron share/exchange between the adsorbent and adsorbate^50^. Moreover, the Elovich model also fitted the data effectively for PNP adsorption onto TMA-(PA6-*g*-GMA) (Table 1). Thus, adsorption can be characterized as a diffusion process with a complex heterogeneous phase, where the adsorbent surface energy was non-uniformly distributed and chemical adsorption took place^51^.

**Table 1:** The parameters of kinetics models for PNP adsorption on TMA-(PA6-*g*-GMA).

PNP initial concentration (mg/L)	20	50	100	150	200
q_exp_ (mg/g)	20.010	41.620	74.950	113.290	126.140
**Pseudo-first order**					
q_cal_ (mg/g)	19.604	39.868	71.779	110.678	120.147
K_1_ (1/min)	0.596	0.288	0.452	0.473	0.392
*R*^2^	0.996	0.973	0.984	0.994	0.978
SSE	1.488	40.184	77.851	65.254	303.443
**Pseudo-second order**					
q_cal_ (mg/g)	19.879	41.744	73.781	112.931	124.3735
K_2_ (g/mg min)	0.124	0.014	0.016	0.0136	0.007
*R*^2^	0.999	0.996	0.995	0.999	0.994
SSE	0.417	6.173	25.277	8.216	80.093
**Elovich**					
a (mg/g min)	2.04 × 10^19^	1.8 × 10^5^	2.77 × 10^9^	1.23 × 10^14^	6.05 × 10^7^
b (g/mg)	2.512	0.380	0.342	0.318	0.167
*R*^2^	0.999	0.995	0.999	0.998	0.999
SSE	0.104	7.980	1.660	13.957	9.029
Intraparticle diffusion model*					
C_ip_ (mg/g)	18.715	33.715	65.016	103.62	106.28
K_ip_ (mg/g min^0.5^)	0.114	0.727	0.859	0.865	1.733
*R*^2^	0.839	0.7613	0.9174	0.7425	0.8844

*Represented the calculated parameters derived from the linear model.

The way adsorption occurred was also essential to establish. The intraparticle diffusion model was adopted for additional analysis of the limited step of the rate of PNP diffusion on TMA-(PA6-*g*-GMA). Four major phases of the adsorption process were distinguished. The first phase involved migration of the adsorbate from the solution bulk to the layer next to the adsorbent. The second and third phases involved diffusion of the molecules through the layer until the adsorbent was reached and through the pores on the adsorbent surface. The fourth phase involved adsorption of the adsorbate by the adsorbent. The process of adsorption was controlled by at least one of these phases, which was identified as the rate-limiting step. When identifying this step, the initial and final phases can be ignored because they are generally rapid^52^. The intraparticle diffusion model was applied with *q*_*t*_ plotted against t^0.5^ to determine whether the adsorption was controlled by the second or third phase. Adsorption is due exclusively to intraparticle diffusion, if a plot line going through the origin is attained, whereas the rate-limiting step encompasses pore diffusion and surface chemical reaction, besides intraparticle diffusion, if the plot does not go through the origin^53^.

Intraparticle diffusion was not the only rate-limiting step in PNP adsorption by TMA-(PA6-*g*-GMA), since the linearised plots (regression lines) of Weber and Morris model did not go through the origin and the *R*^2^ value was less fitted (< 0.9) (Fig. S2d). Hence, adsorption probably depended on multiple processes, encompassing both the surface adsorption and intraparticle diffusion in the adsorption of PNP by TMA-(PA6-*g*-GMA) fibers. Comparable results were reported in previous studies on the use of commercial activated carbon for phenol adsorption and the use of activated carbon derived from desert date seed shell for malachite green adsorption^54^.

#### Adsorption isotherms

To determine how the TMA-(PA6-*g*-GMA) adsorptive capacity and the PNP concentration were correlated in the residual solutions at adsorption equilibrium and to gain insight into the events occurring during PNP adsorption, especially the adsorbent-adsorbate interplay, adsorption isotherms were employed. To that end, the nonlinear Langmuir (Eq. 9), Freundlich (Eq. 10), Temkin (Eq. 11), and Redlich–Peterson (Eq. 12) models were adopted. The premise underpinning the Langmuir model is that the adsorbent surface is uniform and adsorption on it constitutes a monolayer^55^. The separation factor R_L_ was computed to assess whether monolayer adsorption and surface homogeneity were appropriate. Thus, favourable, unfavourable, linear, and irreversible adsorption was respectively indicated by 0 < R_L_ < 1, R_L_ > 1, R_L_ = 1, and R_L_ = 0^56^.

The empirical Freundlich model characterized multilayer adsorption on a non-uniform adsorbent surface. The intensity of adsorption is given by 1/n, with adsorption being favourable, unfavourable or irreversible for 0 < 1/n < 1, l/n > 1, and l/n = 1, respectively^57^.

The adsorbent-adsorbate interplay underpins the Temkin isotherm and is in turn dependent on homogeneous distribution of binding energy, with linear reduction in the adsorption heat as the adsorbate coverage increases. The binding energy and the adsorption heat are respectively denoted by the Temkin constant K_T_ and b_T_. The exothermic or endothermic nature of adsorption is indicated by the b_T_ value; if this value is higher than zero, adsorption is exothermic, with release of heat, whereas if the value is less than zero, adsorption is endothermic, with assimilation of heat^58^.

Integrating the characteristics of Langmuir model and Freundlich model, Redlich–Peterson is an empirical model with three parameters. The exponent is denoted by β and typically has a value in the range from 0 to 1. Langmuir adsorption is reflected by β value near 1, whereas Freundlich adsorption is reflected by β value near 0^57^.

**Figure 11:**
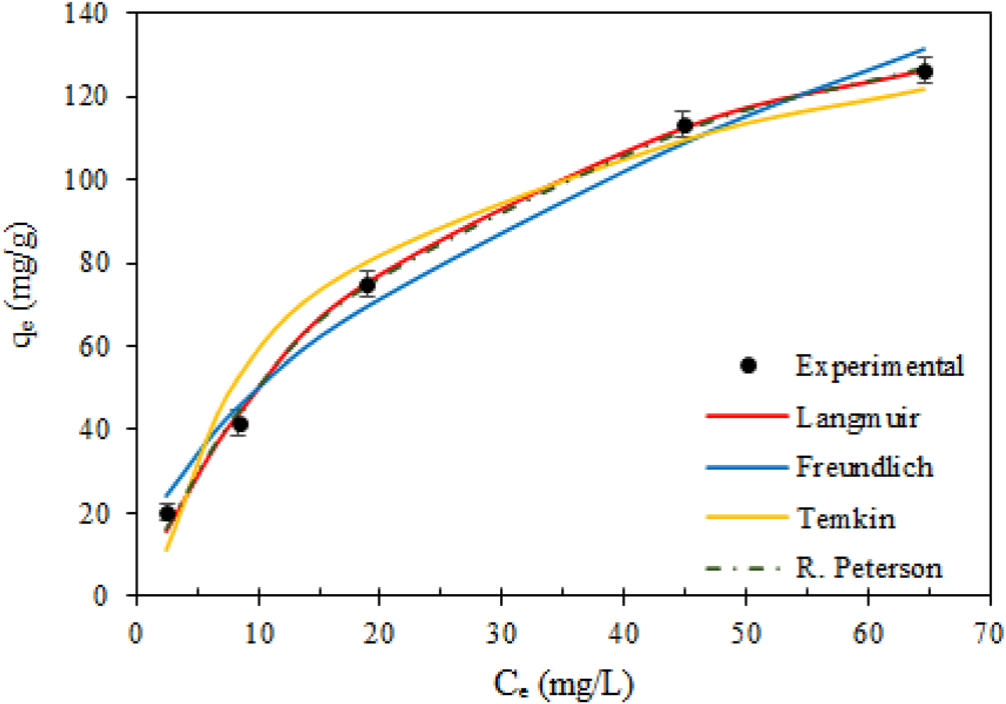
Isotherm models used to fit experimental data for the PNP adsorption on TMA-(PA6-*g*-GMA) fibrous via Langmuir, Freundlich, Temkin, and Redlich Peterson at 298 K, pH 5, adsorbent dose = 0.1 g, volume of solution = 100 mL and agitation speed = 150 rpm.

Figure 11 illustrates isotherm model fitting with equilibrium data for PNP adsorption on TMA-(PA6-*g*-GMA) and the associated isotherm parameters are listed in Table 2. Every isotherm model showed good fitting to the experimental data, since their correlation coefficients were high (0.968–0.997). However, with maximal correlation coefficient *R*^2^ (0.997) and minimal SSE (23.933), the Redlich–Peterson model fitted the data best, indicating that both Langmuir and Freundlich isotherms were incorporated in the adsorption process. The value of the exponent β with in Redlich–Peterson isotherm model is based on Langmuir equation, which has a large *R*^2^ (0.997) and was found to be close to one. Langmuir isotherm had a favourable shape for characterizing the chemical adsorption as the R_L_ values were in the 0–1 range (0.114–0.562). Furthermore, a favourable shape was displayed by the Freundlich model as well, since the l/n reflecting adsorption intensity was in the 0–1 range. Additionally, application of the Temkin model confirmed the exothermic nature of the adsorption process with dissipation of heat at 73.366 J/mol.

**Table 2:** The parameters of isotherm models and equilibrium parameters for PNP adsorption on TMA-(PA6-*g*-GMA).

Isotherm model(s)	Parameters	
Langmuir	q_max_	176.036
	K_L_	0.039
	*R*^*2*^	0.997
	SSE	25.558
Freundlich	K_F_	15.101
	n	1.929
	*R*^*2*^	0.986
	SSE	110.583
Temkin	K	0.571
	b_T_	73.366
	*R*^*2*^	0.968
	SSE	263.700
Redlich, DL Peterson	K_RP_	7.645
	α	0.063
	β	0.917
	*R*^*2*^	0.997
	SSE	23.933
Separation factor (R_L_)	**C**_**ο**_** (mg/L)**	**R**_**L**_
	20	0.562
	50	0.339
	100	0.204
	150	0.146
	200	0.114

#### Thermodynamic functions

Temperatures of between (298 and 318) K were used in the thermodynamic analysis of PNP adsorption by TMA-(PA6-*g*-GMA) fibers. The following formulas were applied to determine the thermodynamic parameters of Gibb’s free energy change (**∆**G°), enthalpy change (**∆**H°) and entropy change (**∆**S°):

18 $$\Delta {\text{G}}^{ \circ } = - {\text{RT}}\ln k_{d} ,$$

19 $$\ln k_{d} = \ln \left[ {\frac{{q_{e} }}{{C_{e} }}} \right] = \frac{{\Delta {\text{S}}^{ \circ } }}{R} - \frac{{\Delta {\text{H}}^{ \circ } }}{RT}.$$

In the above, the temperature (K) is denoted by T, the ideal gas constant of 8.314 J/mol K is denoted by R, and the linear sorption distribution coefficient (q_e_/C_e_) is denoted by *k*_*d*_. The ln *k*_*d*_ was plotted against 1/T based on the Van’t Hoff equation (Figure S3). Table 3 provides the determined thermodynamic parameters. The process was indicated to be feasible and PNP adsorption by TMA-(PA6-*g*-GMA) spontaneous as **∆**G° had negative values. The exothermic character of adsorption was reflected by the negative value of **∆**H°, as well as decreases in PNP removal as the temperature rises (Fig. 10) and the fact that lower temperature allowed greater adsorptive capacity. Furthermore, there was a decline in randomness at the interface between the solid and solution in the adsorption system during the process of adsorption since **∆**S° had a negative value^59^. Similar results were reported in^60^, confirming the exothermic nature of the process of PNP adsorption by nitrogen doped reduced graphene oxide.

**Table 3:** Thermodynamic parameters for PNP adsorption on TMA-(PA6-*g*-GMA).

T (K)	*k*_*d*_	∆G° (kJ/mol)	∆H° (kJ/mol)	∆S° (kJ/mol K)
298	4.818	− 3.896	− 77.198	− 0.247
308	1.366	− 0.799		
318	0.683	1.009		

### Analysis of regeneration potential

When assessing adsorbent efficiency in treating wastewater, it is important to consider reusability. To appraise their cycle performance and the extent to which they could be recycled, TMA-(PA6-*g*-GMA) fibers were washed after use and subsequently used again in fresh experiments. For this purpose, 0.1 M NaOH was employed, with five repetitions of the recycling process. PNP recovery was almost 100% in every cycle. FTIR spectra were compared (Figure S4) but no marked spectral changes were observed following TMA-(PA6-*g*-GMA) regeneration. This suggested that TMA-(PA6-*g*-GMA) was acceptably stable and permitted full regeneration for renewed use without decline in performance. Therefore, the process can be said to possess sustainability and can be deployed in a cost-effective and environmentally friendly way.

### Proposed adsorption mechanism

**Figure 12:**
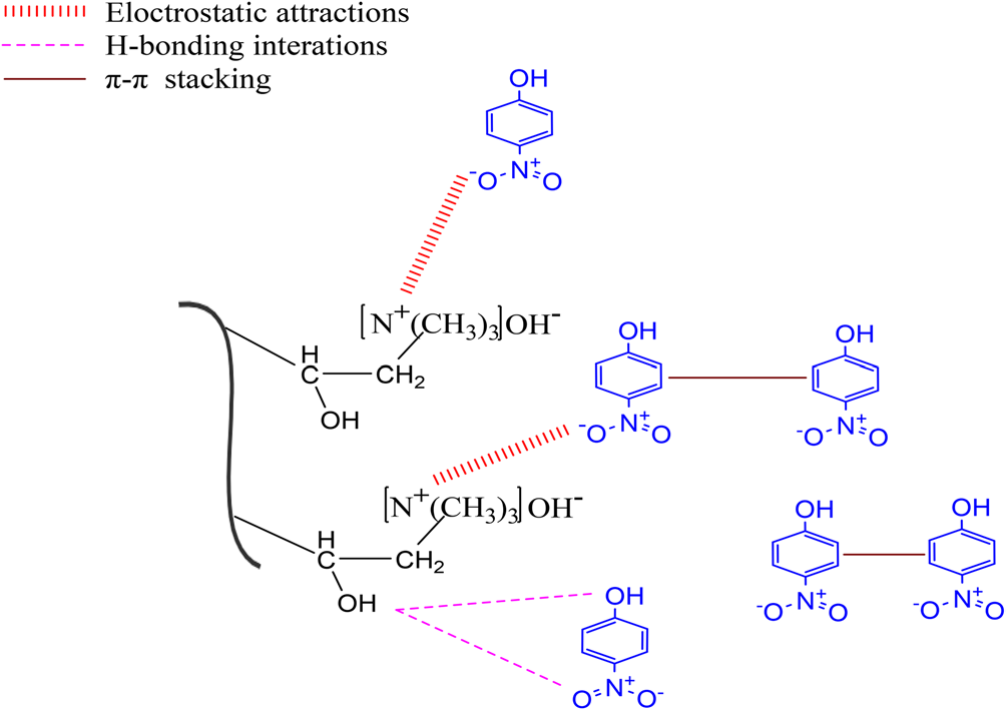
Elucidation of the proposed mechanism for PNP adsorption onto TMA-(PA6-*g*-GMA).

According to the outcomes of the adsorption studies and the instrumental analysis exploring the multi-binding interactions between the fibrous adsorbent and PNP, the mechanism of PNP adsorption on TMA-(PA6-*g*-GMA) was established, as schematically represented in Fig. 12. Affinity and increased availability of binding sites were provided by the greater number of amino and hydroxyl groups in TMA-(PA6-*g*-GMA). The aromatic ring of the PNP molecule encompassed functional groups of single-bond OH and single-bond NO_2_, denoting electron donor and acceptor respectively. The attraction force of greatest impact that could take place between the adsorbent surface and PNP was the electrostatic attraction, whereby amine group with positive charge electrostatically interacted with *p*-nitrophenol negative group (–O)^61^. Moreover, hydrogen bonding occurs between the adsorbent free hydrogen and oxygen within the *p*-nitrophenol structure, whilst the electron-donating groups of oxygen and nitrogen on the surface of the adsorbent and *p*-nitrophenol aromatic ring displayed π–π interaction.

The FTIR spectra of TMA-(PA6-*g*-GMA) and PNP loaded TMA-(PA6-*g*-GMA) (i.e., fibrous adsorbent sample analysed following use in PNP adsorption experiments) were examined to confirm the mechanism of adsorption. Figure S4 shows that the fibrous adsorbent contained functional groups and notable peaks emerged after PNP adsorption. Comparative analysis of the two spectra revealed changes in positions and in band intensity, pointing at the occurrence of interactions during the process of PNP adsorption. It is particularly worth highlighting that there was an increase in the intensity of the peaks at 1733 cm^−1^ and at 1168 cm^−1^, which were attributed to the aromatic C=O bond stretching vibrations and the C–O bond respectively^62^. The FTIR spectra associated with PNP further indicated that the O–H stretching vibration peak determined the band at 3300 cm^−1^, while the NO_2_ group vibration was the reason for the band at 1336 cm^−1^^63^. Moreover, out-of-plane bending =C–H vibration on the benzene ring with –NO_2_ and –OH para-orienting groups determined the band at 857 cm^−1^^64^. These results confirmed the efficiency of PNP adsorption on the fibrous adsorbent.

## Conclusion

An electron beam method was adopted for GMA grafting on PA6 via radiation-induced graft polymerization. The effect of the dose of radiation and the reaction time of grafting on the degree of GMA grafting were investigated. A temperature of 40 °C with optimum absorbed dose of 25 kGy for half an hour were the conditions under which GMA emulsion grafting on PA6 was attained (200%). Subsequently, TMA functionalization was performed on the resulting grafted fibrous material. The grafting and amination reactions were undertaken successfully, as attested by the outcomes of FTIR and FESEM analyses. The TMA-(PA6-*g*-GMA) was examined regarding its ability to remove the phenolic pollutant *p*-nitrophenol from aqueous solution.

The dose of absorbent, pH of PNP solution, temperature, initial PNP concentration, and the residence time were identified as the factors influencing PNP removal by TMA-(PA6-*g*-GMA), with the optimum parameters of adsorption being established to be 0.1 g dose, pH 5, 298 K temperature, and 2-h time. Kinetic analyses revealed the factors underpinning PNP adsorption was described well by Pseudo-second order. Meanwhile, Redlich–Peterson isotherm model provided the best fitting of the data related to TMA-(PA6-*g*-GMA), which the β exponent of Redlich–Peterson model had a value near to one, based on Langmuir isotherm model with its significantly high *R*^2^ of 0.997. The TMA-(PA6-*g*-GMA) exhibited maximum adsorptive capacity of 176.036 mg/g at a temperature of 298 K, according to the isotherm studies. Moreover, the process of PNP adsorption was found to be exothermic and spontaneous in nature, as demonstrated by the thermodynamic analysis. Regeneration of the fibrous adsorbent could also be readily achieved with a low concentration of NaOH and a minimum of five recycling repetitions could be accomplished with no reduction in performance. Therefore, based on the results obtained, it can be said that TMA-(PA6-*g*-GMA) has great potential for use as an adsorbent to adsorb PNP from aqueous solutions.

## Supplementary Information

Supplementary Information.
