# Supplementary material for: Trimethylamine functionalized radiation-induced grafted polyamide 6 fibers for p-nitrophenol adsorption
Source: Sci Rep. 2021 Oct 1;11:19573. doi: 10.1038/s41598-021-97397-y (PMC8486744; doi:10.1038/s41598-021-97397-y)
Supplement: Supplementary file 1 — Supplementary Information. [file 41598_2021_97397_MOESM1_ESM.docx]

**Supplementary Information**

**Trimethylamine Functionalized Radiation-Induced Grafted Polyamide 6 Fibers for *p*-Nitrophenol Adsorption**

Shihab Ezzuldin M.Saber^1,6*^, Luqman Chuah Abdullah^1,2*^, Siti Nurul Ain Md Jamil^3,4^, Thomas S.Y. Choong^1,2^, and Teo Ming Ting^5^

^1^Department of Chemical and Environmental Engineering, Faculty of Engineering, Universiti Putra Malaysia, UPM Serdang 43400, Selangor, Malaysia,

^2^Institute of Tropical Forestry and Forest Products (INTROP), Universiti Putra Malaysia, UPM Serdang 43400, Malaysia,

^3^Department of Chemistry, Faculty of Science, Universiti Putra Malaysia, UPM Serdang 43400, Selangor, Malaysia,

^4^Centre of Foundation Studies for Agricultural Science, Universiti Putra Malaysia, UPM Serdang 43400, Selangor, Malaysia,

^5^Radiation Technology Division, Malaysian Nuclear Agency, 43000, Kajang, Selangor, Malaysia,

^6^North Refineries Company, Baiji, Salahuddin, Ministry of Oil, Iraq.

*****Corresponding author

Shihab Ezzuldin M.Saber

Department of Chemical and Environmental Engineering,

Faculty of Engineering, Universiti Putra Malaysia,

UPM Serdang 43400, Selangor, Malaysia

Email: [shihab.ezzuldeen@gmail.com](mailto:shihab.ezzuldeen@gmail.com)

Professor. Dr. Luqman Chuah Abdullah

Department of Chemical and Environmental Engineering,

Faculty of Engineering, Universiti Putra Malaysia,

UPM Serdang 43400, Selangor, Malaysia

Email: [chuah@upm.edu.my](mailto:chuah@upm.edu.my)


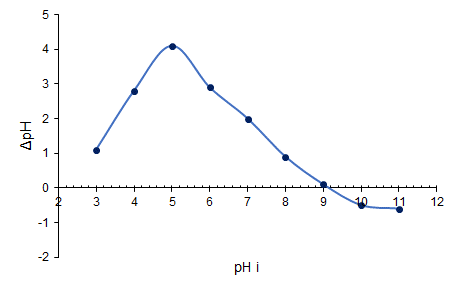


**Figure S1.** pHpzc of TMA-(PA6-*g*-GMA)


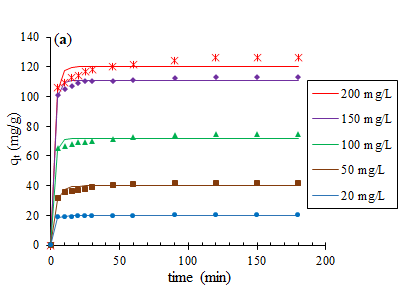


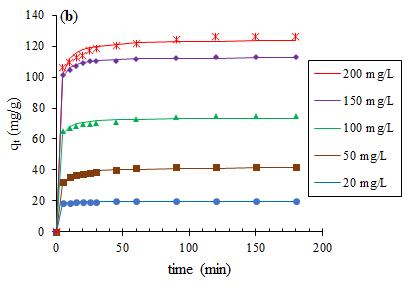


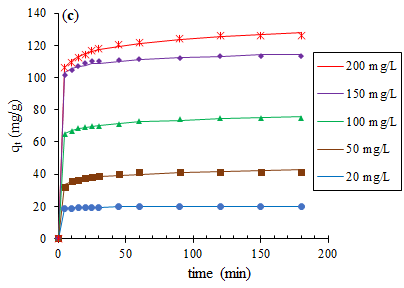


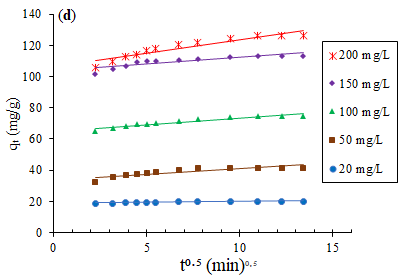


**Figure S2.** Use of various models to fit the kinetic data recorded for the adsorption of PNP onto TMA-(PA6-*g*-GMA) (qt: amount of PNP adsorbed at time t (min) via the nonlinear regression method; (a)PFO, (b) PSO, (c) Elovich, and (d) Intraparticle diffusion linear model, the concentration of PNP was varied (20 - 200) mg/L. The experiments were conducted at 0.1g/100mL as a dosage of fibrous adsorbent, solution pH of 5, the temperature at 25 °C, and agitation speed on 150 rpm. The amount of PNP adsorbed onto TMA-(PA6-*g*-GMA) was measured at the indicated time points.


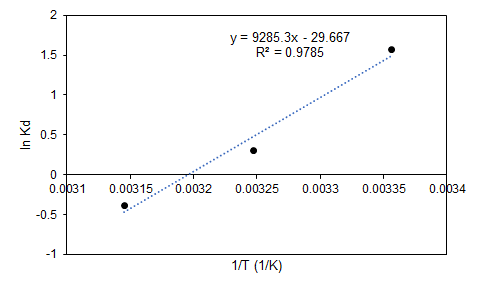


**Figure S3.** Van’t Hoff plot for PNP adsorption onto TMA-(PA6-*g*-GMA) (adsorbent dose = 0.1g, volume of solution = 100 mL, pH 5 and agitation speed = 150 rpm)


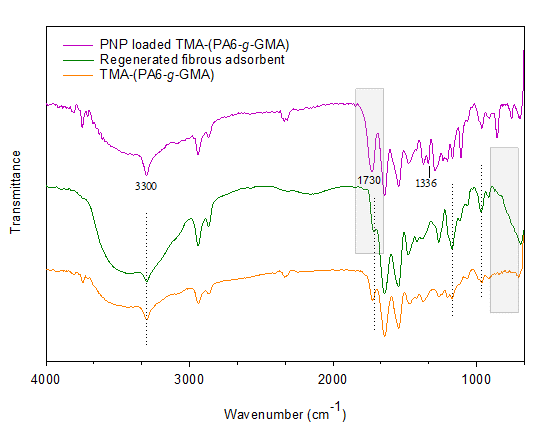


**Figure S4.** FTIR spectra of TMA-(PA6-*g*-GMA), Regenerated TMA-(PA6*-g-*GMA), and PNP loaded TMA-(PA6*-g-*GMA) (fibrous after adsorption)

**Table S1.** BET surface area and pore size for fibrous adsorbent

| **Textural properties** | **PA6** | **TMA-(PA6-*g*-GMA)** |
| --- | --- | --- |
| Specific surface area (m^2^/g) | 2.666 | 1.302 |
| Mean pore volume (cm^3^/g) | 0.293 | 0.283 |
| Mean pore size (nm) | 220 | 435 |
